# The Effects of Positive Psychological Factors on the Mental Wellbeing of Medical Students

**DOI:** 10.7759/cureus.60702

**Published:** 2024-05-20

**Authors:** Alex Collins, Stephanie Stroever, Regina Baronia, Jansen Michaela, Abdul Awal, Jonathan Singer, Wail Amor, Yasin Ibrahim

**Affiliations:** 1 Department of Psychiatry, Texas Tech University Health Sciences Center, Lubbock, USA; 2 Department of Medical Education, Texas Tech University Health Sciences Center, Lubbock, USA; 3 Clinical Research Institute, Texas Tech University Health Sciences Center, Lubbock, USA; 4 Department of Psychological Sciences, Texas Tech University, Lubbock, USA

**Keywords:** medical school curriculum, medical student, mental health, positive psychology, student wellness, medical education

## Abstract

Objective

A well-established association exists between academic performance and levels of depression and anxiety among medical students. However, the effects of positive psychological factors on symptoms of depression and anxiety and academic performance have not been adequately studied. This study explores the relationship between the above variables and identifies positive psychological factors that can promote medical student wellbeing.

Methods

Medical students were surveyed at four time points during their first two years of medical education using Qualtrics. The surveys used a five-point Likert scale to assess students’ levels of loneliness, religiosity, engaged living, life fulfillment, resilience, psychological wellbeing, and symptoms of depression and anxiety. Academic performance was measured using students' Comprehensive Basic Science Examination scores. Linear mixed effect models with maximum likelihood estimation were used to investigate the relationship between positive psychological factors and scores on depression and anxiety as well as the relationship between demographic and psychological factors and exam scores.

Results

Seventy-two students completed the study. A significant positive correlation was observed between loneliness and symptoms of depression and anxiety, while the same symptoms had significant negative correlations with engaged living. None of the positive psychological factors were significantly predictive of exam scores.

Conclusion

Our findings suggest that medical students who develop meaningful relationships and live engaged lives are less likely to develop symptoms of depression and anxiety. Our study lays the groundwork for future research focusing on identifying and implementing pre-clinical curriculum changes aiming to improve medical students' mental health.

## Introduction

Medical schools aim to graduate competent physicians who can care for their patients and advance the field of medicine [1]. During their training, medical students are exposed to highly stressful situations and circumstances, which can increase their likelihood of developing symptoms of depression and anxiety [2,3]. Studies have found that medical students with symptoms of depression and anxiety have higher dropout rates, and higher levels of job dissatisfaction later in their careers [1,4]. Prior research has also demonstrated a negative correlation between academic performance and symptoms of depression and anxiety [5]. However, the associations between positive psychological factors and susceptibility to depression or anxiety among medical students have not been examined.

The founders of positive psychology, Martin Seligman and Mihaly Csikszentmihalyi, proposed that studying the strengths that enable individuals to thrive can result in identifying interventions that improve individuals’ quality of life [6]. Since the publication of their original paper, positive psychological factors have been increasingly recognized as an integral part of mental health care. However, there has been a lack of understanding of the relationship between these factors and academic performance among first- and second-year medical students.

Therefore, in this study, we first aimed to investigate the relationship between positive psychological factors, such as engaged living, resilience, social support, and spirituality, and levels of depression and anxiety. Additionally, we explored the association between positive psychological factors and the academic performance of medical students during their pre-clinical years [7]. By examining the relationships between the above variables during the first two years of medical school, we aimed to identify positive psychological factors that can promote a balance between academic outcomes and the mental wellbeing of first- and second-year medical students.

## Materials and methods

Procedure

The sample consisted of medical students recruited via advertisements placed on bulletin boards within a medical school in West Texas. These bulletin boards were placed in high-traffic campus areas and were accessible to all 174 first-year medical students. Students who chose to participate were provided a link via their official school email to an online consent form and a screening questionnaire on Qualtrics during the first two months of the academic year. Students received a link to each of the four surveys on Qualtrics via email throughout the study (Appendix A). To be eligible to participate, individuals had to be first-year medical students between 18 and 65 years of age. Exclusion criteria included a self-reported current or past history of suicidal ideation or psychiatric disorders and current or past intake of psychiatric medication. Only participants who met the eligibility requirements and provided their consent to participate in the study could proceed to the first survey following the screening questionnaire. Approved participants were required to use the last five digits of their cellphone numbers as a unique subject identification number to mitigate the risk of breaching confidentiality and making it possible to analyze and report only de-identified data.

Four surveys were conducted between August 2020 and April 2022, and only students who completed all four surveys were included in the data analysis (n=63). The first survey (T0) was collected during the first two months of the first academic year (months 1 and 2), the second survey (T1) was collected one year later during the second academic year (months 13 and 14), and the third and fourth surveys (T2 and T3) were collected during the second academic year before (months 18 and 19), and after (months 20 and 21) the National Board of Medical Examiners Comprehensive Basic Sciences Examination (CBSE) was administered, respectively. Performance on the CBSE is considered a measure of preparedness for taking the United States Medical Licensing Exam (USMLE) Step One exam. USMLE Step One assesses the understanding and application of basic science knowledge to medicine. Passing USMLE Step One is required for promotion into the next curricular phase. CBSE scores were gathered from the school’s records to measure academic performance at T3. Participants received a $15 Swift card after completing each of the first three surveys and a $30 Swift card after completing the fourth survey. This study was approved by Texas Tech University Health Sciences Center at Lubbock Institutional Review Board (approval L20-140).

Measures

Symptoms of anxiety and depression were measured by Generalized Anxiety Disorder- 7 (GAD-7) and Patient Health Questionnaire-9 (PHQ-9) validated scales, respectively [8,9]. PHQ-9 is a nine-item measure that assesses depression severity and has shown high internal consistency (a=.89) and test-retest reliability (a=.84) [8]. Total scores of 0-4, 5-9, 10-14, 15-20, and 20-27 represent minimal, mild, moderate, moderately severe, and severe depression, respectively.

GAD-7 is a seven-item self-report scale used to measure the severity of anxiety symptoms, and it is a reliable screening tool for anxiety disorders [9]. GAD-7 exhibits excellent internal consistency (a=.92) and good convergent validity when compared to the Beck Anxiety Inventory (r=.72) [9]. Total scores of 0-4, 5-9, 10-14, and 15-21 represent minimal, mild, moderate, and severe anxiety, respectively.

Loneliness was measured using the De Jong Gierveld Loneliness Scale (ELSL) [10]. ELSL includes two subscales assessing emotional and social loneliness, which indicate the lack of intimate relationships and social networks, respectively. Responses were summed with possible total scores ranging from 0 to 6, with higher scores indicating greater loneliness.

Religiousness and spirituality were measured using The Duke University Religious Index with a Cronbach’s alpha greater than 0.70 [11]. It is a five-item measure of religious involvement that assesses three significant dimensions of religiosity: organizational religious activity (ORA), non-organizational religious activity (NORA), and intrinsic religiosity (IR). Total ORA and NORA scores ranged from 0-6, while IR total scores ranged from 1-15.

The Engaged Living Scale (ELS) was used to measure engaged living and its two subfactors: valued living and life fulfillment [12]. ELS consists of 10 items measuring valued living and six measuring life fulfillment. Responses were summed with possible total scores ranging from 16 to 80, with a higher score indicating a more developed engaged response style.

Resilience is one’s ability to overcome stressful events and successfully face adversity. Resilience was measured using a Connor-Davidson 10-Item Resilience Scale (CDRF) with a Cronbach’s alpha greater than 0.80 [13]. Responses were summed with possible total scores ranging from 0 to 40, with higher scores indicating greater resilience.

Data analysis

Stata version 17.0 (StataCorp LLC, College Station, TX, USA) and RStudio version 2023.09.1+494 (R Foundation for Statistical Computing, Vienna, Austria) were utilized for data management and statistical analyses. Listwise deletion was applied for missing data, and descriptive statistics were computed, including frequency with percentages for categorical variables and mean (standard deviation) or median (interquartile range) for continuous variables. Statistical significance was determined at an alpha level of 0.05.

We used linear mixed effect models with maximum likelihood estimation to investigate the relationship between positive psychological factors and scores on depression and anxiety. This approach was chosen to accommodate the hierarchical structure of the dataset where observations were clustered within individuals over different time points (T0, T1, T2, and T3). The outcomes were modeled in two separate analyses, and we included age, gender, ethnicity, and positive psychological factors (ELSL, ORA, NORA, IR, ELS, CDRF) as fixed effects and the participant as a random effect. The data underwent thorough visual inspections to assess normality and homogeneity of distributions, and all assumptions of the linear mixed effect models were verified prior to interpretation.

Additionally, we used a linear mixed-effect model to analyze the relationship between demographic and psychological factors and CBSE scores to assess the association between psychological factors and the academic performance of medical students. Fixed and random effects were included as described above. Lastly, we employed separate models to assess the effects of depression and anxiety on CBSE scores by including PHQ-9 and GAD-7 scores as fixed effects. We conducted the analyses separately for each scale and modeled the effects on both the continuous scale and as dichotomous variables. The dichotomous scales represented no score on depression (PHQ-9 = 0) compared to any score on depression (PHQ-9 > 0) and no score on anxiety (GAD-7 = 0) compared to any score on anxiety (GAD-7 > 0).

In the linear mixed-effects model, fixed effects were the average effect of the predictors on the response variable at all levels of random effects while the random effects captured variation at various levels of grouping in the dataset (Appendix B). This approach was chosen to accommodate the hierarchical structure of the dataset, where observations were clustered within individuals over different time points (T0, T1, T2, and T3). This modeling approach allowed us to account for both fixed effects, representing systematic influences like demographic and psychological factors, and random effects, capturing individual-specific variations over different time points. The model incorporated depression and anxiety levels as outcome variables. Fixed effects included predictors such as age, gender, ethnicity, and positive psychological scales while individuals and the different time points were considered random effects.

## Results

One hundred and six of the 174 (99 female and 75 male) first-year medical students responded to the invitation to participate in the study, and the response rate was 60.91%. Thirty-four out of the 106 met one or more exclusion criteria. A total of 72 students completed the baseline survey, and 86.3% of the enrolled students (n=63) completed surveys at all four time points. The sample was predominantly female (n=48; 75.86%) and white (n=38; 59.51%), while 18 students identified as Asian (29.15%), three identified as African American (4.86%), and four identified as Other (6.48%). The mean age was 23.67 (SD=2.46), ranging from 21 to 37.

The mean PHQ-9 and GAD-7 scores indicated minimal depression at all time points and mild anxiety at T0 and T3. Both PHQ-9 and GAD-7 scores significantly varied between individuals, with standard deviations of 2.14 (95% CI [1.538, 2.483]) and 2.76 (95% CI [2.073, 3.185]), respectively. The PHQ-9 scores exhibited minimal variability with time (SD=0.3, 95% CI [0.0, 0.933]), while GAD-7 scores demonstrated significant differences between time points (SD=0.688, 95% CI [0.249, 1.688]).

Loneliness (ELSL) demonstrated a significant positive effect on PHQ-9 (p < 0.05), with a 0.48 increase in PHQ-9 scores for every unit of ELSL score increase (Table 1). Conversely, engaged living (ELS) exhibited a significant negative effect (p < 0.05), indicating that a unit change in ELS score is linked to lower PHQ-9 scores by 0.1 units. Neither resilience (CDRF) nor religiousness and spirituality (ORA, NORA, and IR) were significantly correlated with symptoms of depression.

**Table 1:** Summary of the Effects of Various Predictors on PHQ-9 Scores Abbreviations: PHQ-9, Patient Health Questionnaire-9; ∆, change per one unit increase; SE, Standard Error; CI, Confidence Interval; PPFs, Positive Psychological Factors; ELSL, De Jong Gierveld Loneliness Scale; ORA, organizational religious activity; NORA, non-organizational religious activity; IR, intrinsic religiosity; ELS, Engaged Living Scale; CDRF, Connor-Davidson 10-Item Resilience Scale *P-value < 0.05

	PHQ-9			
	∆	SE	p-value	95% CI
Age	0.263	0.145	0.075	(-0.008, 0.532)
Sex				
Male (Reference)	-	-	-	-
Female	1.329	0.770	0.089	(-0.108, 2.752)
Race/ethnicity				
White (Reference)	-	-	-	-
African American	0.932	1.527	0.543	(-1.915, 3.762)
Asian	-1.118	0.716	0.124	(-2.455, 0.205)
Other Ethnicities	-2.606	1.357	0.059	(-5.128, -0.087)
PPFs				
ELSL	0.482	0.148	< 0.05*	(0.203, 0.773)
ORA	0.166	0.226	0.464	(-0.262, 0.597)
NORA	-0.062	0.179	0.729	(-0.398, 0.291)
IR	-0.011	0.105	0.913	(-0.225, 0.182)
ELS	-0.102	0.038	< 0.05*	(-0.177, -0.028)
CDRF	-0.067	0.058	0.252	(-0.181, 0.044)

Loneliness (ELSL) showed a significant positive effect on GAD-7 (p < 0.05), with a 0.49 increase in GAD-7 scores for every unit of ELSL score increase (Table 2), while engaged living (ELS) had a significant negative effect (p < 0.05) with a 0.13 decrease in GAD-7 for every one-unit change in ELS. Neither resilience (CDRF) nor religiousness and spirituality (ORA, NORA, and IR) were significantly correlated with symptoms of anxiety.

**Table 2:** Summary of the Effects of Various Predictors on GAD-7 Scores Abbreviations: GAD-7, Generalized Anxiety Disorder-7; ∆, change per one unit increase; SE, Standard Error; CI, Confidence Interval; PPFs, Positive Psychological Factors; ELSL, De Jong Gierveld Loneliness Scale; ORA, organizational religious activity; NORA, non-organizational religious activity; IR, intrinsic religiosity; ELS, Engaged Living Scale; CDRF, Connor-Davidson 10-Item Resilience Scale *P-value < 0.05

	GAD-7			
	∆	SE	p-value	95% CI
Age	0.128	0.178	0.475	(-0.206, 0.461)
Sex				
Male (Reference)	-	-	-	-
Female	1.217	0.950	0.205	(-0.560, 2.992)
Race/ethnicity				
White (Reference)	-	-	-	-
African American	-0.774	1.876	0.681	(-4.287, 2.729)
Asian	-1.089	0.883	0.222	(-2.746, 0.556)
Other Ethnicities	-2.433	1.679	0.152	(-5.566, 0.707)
PPFs				
ELSL	0.486	0.163	< 0.05*	(0.178, 0.807)
ORA	-0.058	0.253	0.818	(-0.548, 0.423)
NORA	0.154	0.194	0.426	(-0.209, 0.546)
IR	-0.017	0.116	0.882	(-0.244, 0.203)
ELS	-0.128	0.042	< 0.05*	(-0.210, -0.046)
CDRF	-0.042	0.064	0.513	(-0.167, 0.080)

None of the positive psychological factors were significantly predictive of CBSE scores (Table 3). Furthermore, our data demonstrated no correlation between academic performance and levels of depression and anxiety when modeled with both continuous and dichotomous variables (Table 4).

**Table 3:** Summary of the Effects of Various Predictors on CBSE Scores Abbreviations: CBSE, National Board of Medical Examiners Comprehensive Basic Sciences Examination; ∆, change per one unit increase; SE, Standard Error; CI, Confidence Interval; PPFs, Positive Psychological Factors; ELSL, De Jong Gierveld Loneliness Scale; ORA, organizational religious activity; NORA, non-organizational religious activity; IR, intrinsic religiosity; ELS, Engaged Living Scale; CDRF, Connor-Davidson 10-Item Resilience Scale *P-value < 0.05

	∆	SE	p-value	95% CI
Age	-0.414	0.519	0.425	(-1.434, 0.604)
Sex				
Male (Reference)	-	-	-	-
Female	-2.289	2.727	0.401	(-7.63, 3.056)
Race/ethnicity				
White (Reference)	-	-	-	-
African American	-3.070	5.494	0.576	(-13.839, 7.698)
Asian	-3.395	2.554	0.184	(-8.403, 1.611)
Other Ethnicities	5.535	4.844	0.253	(-3.959, 15.030)
PPFs				
ELSL	0.113	0.523	0.829	(-0.912, 1.139)
ORA	-0.154	0.791	0.854	(-1.706, 1.397)
NORA	-0.361	0.644	0.575	(-1.623, 0.900)
IR	0.159	0.367	0.664	(-0.560, 0 .879)
ELS	-0.148	0.137	0.279	(-0.417, 0 .120)
CDRF	0.081	0.212	0.703	(-0.335, 0 .497)

**Table 4:** Summary of the Effects of Depression and Anxiety Levels on CBSE Scores Abbreviations: CBSE, National Board of Medical Examiners Comprehensive Basic Sciences Exam Examination; PHQ-9 All, Patient Health Questionnaire-9 All Scores; PHQ-9>0, All, Patient Health Questionnaire-9 Score above 0; GAD-7 All, Generalized Anxiety Disorder-7 All Scores; GAD-7 >0, Generalized Anxiety Disorder-7 Scores Above 0; ∆, change per one unit increase; SE, Standard Error; CI, Confidence Interval *P-value < 0.05

	∆	SE	p-value	95% CI
PHQ-9 All	-0.027	0.132	0.838	(-0.286, 0.232)
PHQ-9 >0	0.295	1.006	0.769	(-1.678, 2.268)
GAD-7 All	-0.19	0.116	0.869	(-0.246, 0.208)
GAD-7 >0	-0.101	0.975	0.917	(-2.013, 1.810)

## Discussion

Our findings suggest that loneliness may contribute to the development of symptoms of depression and anxiety without affecting the academic performance of first- and second-year medical students. Wolf found that medical students often complain about decreased time and opportunity to maintain relationships due to the increased academic demand associated with medical education [14]. Thus, wellness programs that aim to increase social interactions and strengthen social bonds could improve medical students’ mental health. Another finding is that engaged living may play a protective role against depression and anxiety but does not affect medical students’ performances on standardized exams. This suggests that implementing programs into medical curriculums that facilitate conversations about engaging in valued behaviors could foster better mental health among medical students. Fortunately, most US medical schools’ pre-clinical curricula and the USMLE Step exam use a pass-fail scoring system, which can pave the way for curricular reforms. For example, to enhance student's learning experience, schools should consider replacing a few pre-clinical lectures with longitudinal electives. Students could explore their interests through these electives and participate in research or community outreach projects. These activities would allow students to engage in value-congruent activities while building connections with their peers, faculty, and community [15].

Surprisingly, results from the present study did not identify a significant relationship between spirituality and symptoms of depression and anxiety. This result contradicts previous studies reporting a negative association between these two factors [16,17]. The study was conducted during the coronavirus disease 2019 (COVID-19) pandemic, thus the inconsistency may be due to the limited access to religious and spiritual activities, which could decrease their protective effects.

Contrary to our hypothesis, our study showed no significant correlation between positive psychological factors and academic performance. In addition, our findings were inconsistent with previous studies, as no significant relationship was found between neither resilience nor academic performance and levels of depression or anxiety [5,7,18-21]. These findings are likely due to the small sample size of medical students surveyed. Additionally, students with higher levels of anxiety and depression either chose not to participate, did not complete all four surveys, or were excluded from the study, which would explain the low mean PHQ-9 and GAD-7 scores reported by participants.

This study demonstrates several strengths. Firstly, by employing longitudinal data collection at four time points over the course of two years of medical education, it effectively captures changes in psychological factors and symptoms of depression and anxiety over time, enhancing the validity of the findings. Secondly, the use of advanced statistical techniques such as linear mixed effect models with maximum likelihood estimation enables robust analysis, while controlling for potential confounding variables. Moreover, this study offers valuable insights into strategies for promoting both medical education and the overall wellbeing of medical students.

The present study has several limitations. First, only 72 medical students participated; thus, the small sample size decreased the power of the study. Second, the study was conducted in a US medical school, so generalizations for other regions should be made with caution. Third, students with a current or history of suicidal ideation, psychiatric diagnosis, and psychiatric medication use were excluded from the study, making the findings less generalizable. Fourth, the study was conducted during a global pandemic, which may have altered the effects of positive psychological factors. Additional limitations of this study include selection bias introduced by voluntary participation and that positive psychological factors, symptoms of depression, and anxiety were all self-reported, which may be inconsistent with actual behavioral displays.

## Conclusions

Our findings suggest that medical students who develop meaningful relationships or live engaged lives are less likely to develop symptoms of depression and anxiety, but these factors do not significantly impact their academic performance. Thus, curriculum changes that aim to increase the number of incorporated activities targeting the above-mentioned positive psychological factors could improve medical students’ mental health. Also, medical schools are urged to provide mandatory wellness workshops and promote peer support networks to mitigate feelings of isolation and loneliness. Our study lays the groundwork for future research focusing on pre-clinical curriculum changes aiming to improve student wellbeing by encouraging togetherness, teamwork, value clarity, and taking purposeful actions.

## Figures and Tables

**Figure 1:**
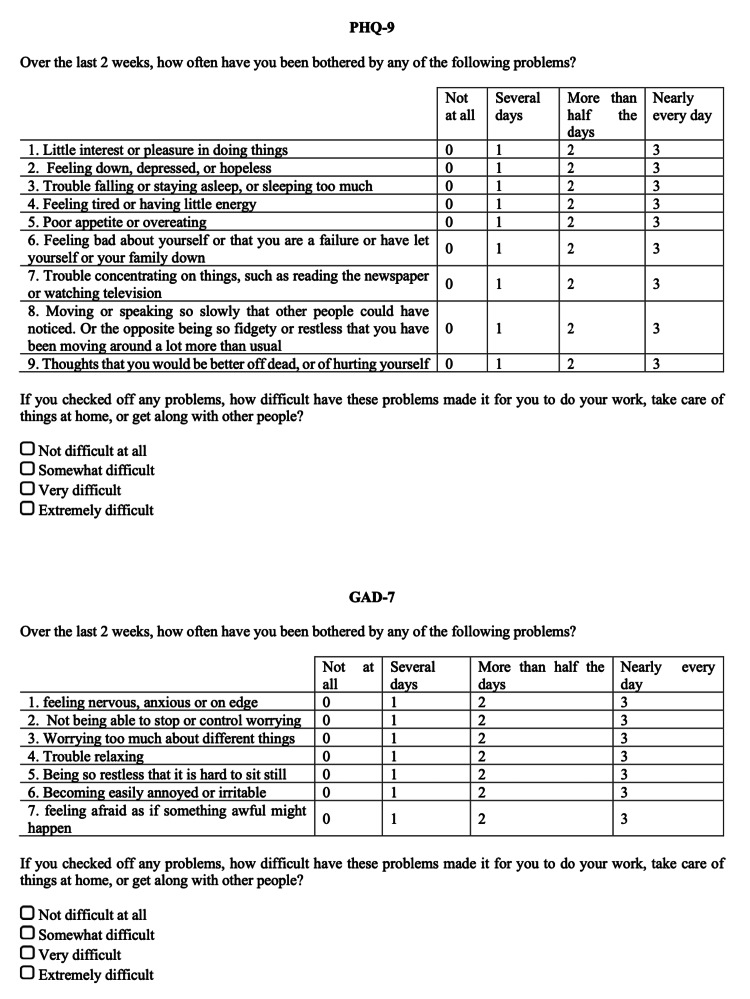
Patient Health Questionnaire-9 (PHQ-9) and Generalized Anxiety Disorder-7 (GAD-7)

**Figure 2:**
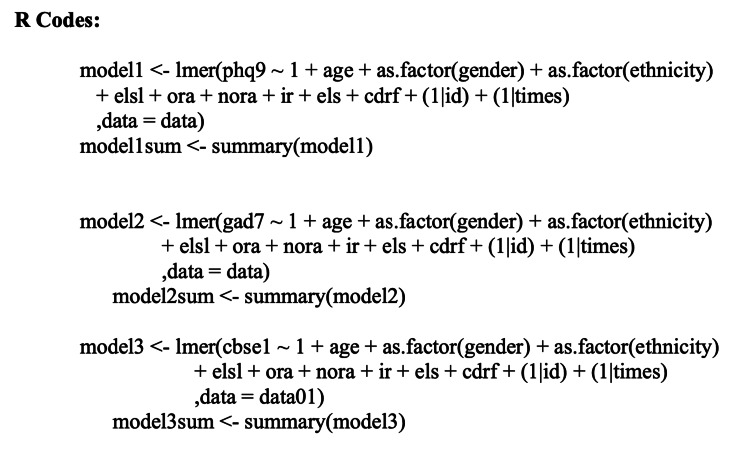
R Codes Abbreviations: PHQ-9, Patient Health Questionnaire-9; GAD-7, Generalized Anxiety Disorder-7; CBSE, National Board of Medical Examiners Comprehensive Basic Sciences Examination; ELSL, De Jong Gierveld Loneliness Scale; ORA, organizational religious activity; NORA, non-organizational religious activity; IR, intrinsic religiosity; ELS, Engaged Living Scale; CDRF, Connor-Davidson 10-Item Resilience Scale

**Figure 3:**
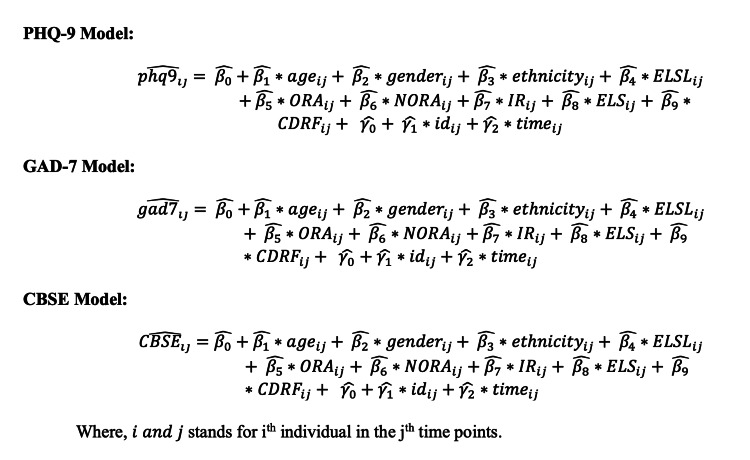
PHQ-9, GAD-7 and CBSE Models Abbreviations: PHQ-9, Patient Health Questionnaire-9; GAD-7, Generalized Anxiety Disorder-7; CBSE, National Board of Medical Examiners Comprehensive Basic Sciences Examination; ELSL, De Jong Gierveld Loneliness Scale; ORA, organizational religious activity; NORA, non-organizational religious activity; IR, intrinsic religiosity; ELS, Engaged Living Scale; CDRF, Connor-Davidson 10-Item Resilience Scale

## Appendices

Appendix A

Screening Questionnaire

Are you currently a first-year medical student for the academic year of 2020-2021?

0 - Yes          1 - No

Are you between the ages of 18-65 years?      

0 - Yes          1 - No

Do you have any current or past history of suicidal ideation?

0 - Yes          1 - No

Do you have any current or past history of any psychiatric condition(s)?

0 - Yes          1 - No

Have you taken or been prescribed psychiatric medications before?

0 - Yes          1 - No

Demographic Questions

Age: __________

Gender

0 - Male

1 - Female 

2 - Other

Ethnicity

0 - White

1 - Black

2 - Asian

3 - American Indian/ Alasan native   

4 - Native Hawaiian/ Pacific Islander

5 - other

6 - no reply

Duke University Religion Index

How often do you attend church or other religious meetings? (ORA)

1 - Never

2 - Once a year or less

3 - A few times a year

4 - A few times a month

5 - Once a week

6 - More than once/week

How often do you spend time in private religious activities, such as prayer, meditation or Bible study?

1 - Rarely or never

2 - A few times a month

3 - Once a week         

4 - Two or more times/week

5 - Daily

6 - More than once a day

The following section contains 3 statements about religious belief or experience. Please mark the extent to which each statement is true or not true for you.

In my life, I experience the presence of the Divine (i.e., God)

1 - Definitely not true

2 - Tends not to be true

3 - Unsure      

4 - Tends to be true

5 - Definitely true of me

My religious beliefs are what really lie behind my whole approach to life

1 - Definitely not true

2 - Tends not to be true

3 - Unsure      

4 - Tends to be true

5 - Definitely true of me

I try hard to carry my religion over into all other dealings in life

1 - Definitely not true

2 - Tends not to be true

3 - Unsure      

4 - Tends to be true

5 - Definitely true of me

Connor-Davidson 10-Item Resilience Scale

Please answer the following questions related to your outlook on life.

I am able to adapt to change.

0 - Not True at All

1 - Rarely True

2 - Sometimes True      

3 - Often True

4 - True Nearly All of the Time 

I can deal with whatever comes my way.

0 - Not True at All

1 - Rarely True

2 - Sometimes True      

3 - Often True

4 - True Nearly All of the Time 

I see the humorous side of things.

0 - Not True at All

1 - Rarely True

2 - Sometimes True      

3 - Often True

4 - True Nearly All of the Time 

I believe coping with stress strengthens me.

0 - Not True at All

1 - Rarely True

2 - Sometimes True      

3 - Often True

4 - True Nearly All of the Time 

I tend to bounce back after illness or hardship.

0 - Not True at All

1 - Rarely True

2 - Sometimes True      

3 - Often True

4 - True Nearly All of the Time 

I believe I can achieve my goals.

0 - Not True at All

1 - Rarely True

2 - Sometimes True      

3 - Often True

4 - True Nearly All of the Time 

Under pressure, I can focus and think clearly.

0 - Not True at All

1 - Rarely True

2 - Sometimes True      

3 - Often True

4 - True Nearly All of the Time 

I am not easily discouraged by failure.

0 - Not True at All

1 - Rarely True

2 - Sometimes True      

3 - Often True

4 - True Nearly All of the Time 

I think of myself as a strong person.

0 - Not True at All

1 - Rarely True

2 - Sometimes True      

3 - Often True

4 - True Nearly All of the Time 

I can handle unpleasant feelings.

0 - Not True at All

1 - Rarely True

2 - Sometimes True      

3 - Often True

4 - True Nearly All of the Time 

Engaged Living Scale

I have values that give my life more meaning.

0 - Completely Disagree

1 - Disagree

2 - Neutral      

3 - Agree

4 - Completely Agree

I know what motivates me in life.

0 - Completely Disagree

1 - Disagree

2 - Neutral      

3 - Agree

4 - Completely Agree

I believe that I’ve found important values to live according to.

0 - Completely Disagree

1 - Disagree

2 - Neutral      

3 - Agree

4 - Completely Agree

I know exactly what I want to do with my life.

0 - Completely Disagree

1 - Disagree

2 - Neutral      

3 - Agree

4 - Completely Agree

I make choices based on my values, even if it is stressful.

0 - Completely Disagree

1 - Disagree

2 - Neutral      

3 - Agree

4 - Completely Agree

I know how I want to live my life.

0 - Completely Disagree

1 - Disagree

2 - Neutral      

3 - Agree

4 - Completely Agree

I know what I want to do with my life.

0 - Completely Disagree

1 - Disagree

2 - Neutral      

3 - Agree

4 - Completely Agree

I believe that my values are really reflected in my behavior.

0 - Completely Disagree

1 - Disagree

2 - Neutral      

3 - Agree

4 - Completely Agree

I believe that how I behave fits in with my personal wants and desires

0 - Completely Disagree

1 - Disagree

2 - Neutral      

3 - Agree

4 - Completely Agree

My emotions don’t hold me back from doing what’s important to me.

0 - Completely Disagree

1 - Disagree

2 - Neutral      

3 - Agree

4 - Completely Agree

I live the way I always intended to live.

0 - Completely Disagree

1 - Disagree

2 - Neutral      

3 - Agree

4 - Completely Agree

I am satisfied with how I live my life.

0 - Completely Disagree

1 - Disagree

2 - Neutral      

3 - Agree

4 - Completely Agree

Nothing can stop me from doing something that’s important to me.

0 - Completely Disagree

1 - Disagree

2 - Neutral      

3 - Agree

4 - Completely Agree

I believe that I am living life to the full right now.

0 - Completely Disagree

1 - Disagree

2 - Neutral      

3 - Agree

4 - Completely Agree

I make time for the things that I consider important.

0 - Completely Disagree

1 - Disagree

2 - Neutral      

3 - Agree

4 - Completely Agree

I feel that I am living a full life.

0 - Completely Disagree

1 - Disagree

2 - Neutral      

3 - Agree

4 - Completely Agree

DeJong Gierveld Loneliness Scale

I experience a general sense of emptiness.

1 - Yes

1 - More or Less

0 - No

I miss having people around me.

1 - Yes

1 - More or Less

0 - No

I often feel rejected.

1 - Yes

1 - More or Less

0 - No

There are plenty of people I can rely on when I have problems.

0 - Yes

1 - More or Less

1 - No

There are many people I can trust completely.

0 - Yes

1 - More or Less

1 - No

There are enough people I feel close to.

0 - Yes

1 - More or Less

1 - No

Appendix B
